# Production and characterization of human tau protein with site‐specific phosphorylation utilizing *E. coli* with an expanded genetic code

**DOI:** 10.1002/alz.091426

**Published:** 2025-01-03

**Authors:** Erik J Ammermann, Yongku P Cho

**Affiliations:** ^1^ University of Connecticut, Storrs, CT USA

## Abstract

**Background:**

With insight into the elevated levels of phosphorylation of diseased tau, it is believed that specific modifications occur in a time‐dependent manner that contribute to tau’s role in Alzheimer’s disease pathogenesis and progression. Present methods to obtain phospho‐tau (p‐tau) from post‐mortem tissue or recombinantly are insufficient to answer the foremost questions in the field, and there is currently no way to study each disease‐relevant modification reproducibly or in isolation. To this point, learning about tau phosphorylation at the resolution of a single modification has been a major obstacle in clarifying whether certain sites are causative of disease or just a by‐product of other harmful mechanisms. Furthermore, a standardized p‐tau reagent with known, consistent modifications is urgently needed to develop diagnostic and phospho‐specific tools.

**Method:**

Using a phosphoserine orthogonal translation system, we describe a method to produce full‐length human tau in engineered *E. coli* with genetically defined phosphoserine (pSer) sites. This p‐tau can be readily purified with traditional chromatography techniques and subsequently used for *in vitro* assays to assess differences in tau biochemistry due to individual PTMs for the first time. To date, we have produced 12 different p‐tau variants with a single disease‐relevant pSer site ranging from pSer199 to pSer422.

**Result:**

In experiments to measure tau aggregation *in vitro* (thioflavin‐T and HEK cell biosensor), our preliminary results indicate that the disease‐associated pSer396 modification of 2N4R tau may have increased aggregation and seeding capabilities relative to other tested pSer tau. Strikingly, in the thioflavin‐T experiment, spontaneous aggregation of pSer396 was observed in which no heparin was added to the reaction mixture. Also, using Western blot, we clearly demonstrate a simple but powerful method to assess the specificity of phospho‐specific antibodies.

**Conclusion:**

The ability to readily produce recombinant human tau with known PTMs will be a critical tool in helping researchers develop, validate, and assess the specificity of p‐tau detection platforms and antibody‐based technologies. With the idea of developmentally significant PTM sites in mind, we have preliminary results suggesting that even single tau PTMs can dramatically alter tau biochemistry and lead to disease‐like molecular behaviors.

